# Total Flavonoids of Drynariae Rhizoma Prevent Bone Loss Induced by Hindlimb Unloading in Rats

**DOI:** 10.3390/molecules22071033

**Published:** 2017-06-22

**Authors:** Shuanghong Song, Ziyang Gao, Xujun Lei, Yinbo Niu, Yuan Zhang, Cuiqin Li, Yi Lu, Zhezhi Wang, Peng Shang

**Affiliations:** 1 Key Laboratory for Space Bioscience and Biotechnology, Institute of Special Environmental Biophysics, School of Life Sciences, Northwestern Polytechnical University, Xi’an 710072, China; songshuanghong@snnu.edu.cn (S.S.); ziyangg@hotmail.com (Z.G.); leixujun163@163.com (X.L.); niuyinbo@yeah.net (Y.N.); luyi@nwpu.edu.cn (Y.L.); 2 Key Laboratory of Ministry of Education for Medicinal Resources and Natural Pharmaceutical Chemistry, National Engineering Laboratory for Resource Developing of Endangered Chinese Crude Drugs in Northwest of China, College of Life Sciences, Shaanxi Normal University, Xi’an 710062, China; yuanzhang@snnu.edu.cn (Y.Z.); licuiqin16@snnu.edu.cn (C.L.); zzwang@snnu.edu.cn (Z.W.)

**Keywords:** traditional chinese medicine, Drynariae Rhizoma, flavonoids, hindlimb unloading, osteoporosis

## Abstract

Drynariae Rhizoma is a kidney-tonifying herb that has a long history in clinical practice for the treatment of bone fractures and joint diseases in China. Flavonoids are considered to be its major active ingredients and are reported to ease bone loss in ovariectomized rats. However, the beneficial effects of the total flavonoids of Drynariae Rhizoma on osteoporosis caused by microgravity or mechanical inactivity remain unknown. This study assessed the effects of total Drynariae Rhizoma flavonoids (DRTF, Qihuang, Beijing, China, national medicine permit No. Z20030007, number of production: 04080081, content of DRTF ≥80%) against bone loss induced by simulated microgravity. A hindlimb unloading tail-suspended rat model was established to determine the effect of DRTF on bone mineral density (BMD), biomechanical strength and trabecular bone microarchitecture. Twenty-eight male Sprague–Dawley rats were divided into four groups: the baseline, control, hindlimb unloading with vehicle (HLU), and hindlimb unloading treated with DRTF (HLU–DRTF, 75 mg/kg/day) groups. Oral DRTF was administered for 4 weeks. The underlying mechanisms of the DRTF actions on disuse-induced osteoporosis are discussed. The results showed that DRTF treatment significantly increased the BMD and mechanical strength of tail-suspended rats. Enhanced bone turnover markers with HLU treatment were attenuated by DRTF administration. Deterioration of trabecular bone induced by HLU was prevented through elevated bone volume/tissue volume (BV/TV), trabecular number (Tb. N), trabecular thickness (Tb. Th) and decreased trabecular separation (Tb. Sp). The present study provides the first evidence that DRTF prevents bone loss induced by HLU treatment, indicating its potential application in the treatment of disuse-induced osteoporosis.

## 1. Introduction

Mechanical loading is critical for the maintenance of skeletal integrity. Unloading during immobilization, space flight or prolonged bed rest interrupts the bone homeostasis between bone formation and bone resorption, leading to various disorders, such as bone loss [1], muscle atrophy [2], immune function decline [3,4], and neuropsychiatric symptoms [5]. After 17 weeks of horizontal bed rest, a 3% and 9% decrease of total hip bone mineral density (BMD) and calcaneus BMD were observed, respectively, in 18 control subjects who followed the same bed rest protocol without exercise. Among astronauts and cosmonauts who participated in long duration flights aboard Mir space station (Mir) and International space station (ISS), >50% of the crew members had a ≥ 10% loss in at least one skeletal site, and 22% of the Mir cosmonauts had a 15%–20% loss in at least one site [6,7]. Disuse-induced osteoporosis not only threatens the safety and health of astronauts during space flight, but also increases the susceptibility to fractures in patients and the elderly requiring bed rest. It is essential to identify relevant countermeasures, such as drug intervention, strengthening exercises [8], and nutrition [9] to reduce or prevent such bone loss.

A number of traditional herbal medicines or natural compounds show preventative effects on bone loss induced by physical inactivity or estrogen deficiency, such as icaritin [10,11], resveratrol [12,13], astragaloside II [14], epimedium [15], Radix Dipsaci [16] and *Eucommia ulmoides* [17]. These natural products have gained increasing attention and have become an important resource for new drug discovery in recent years. Drynariae Rhizoma is the dried rhizome of the perennial pteridophyte *Drynaria fortunei* (Kunze) J. Sm. It is officially listed in the Chinese Pharmacopoeia and is traditionally used for the treatment of bone fractures or related diseases. Its Chinese name is “Gu sui bu” which means “bone fractures healer”. Rhizoma drynariae was first recorded in <BEN CAO SHI YI> of the Tang dynasty by Chen Z.Q. over 1000 years ago [18]. However, modern pharmacology research of Rhizoma drynariae began in the early 1990s [18,19]. The crude extracts of Drynariae Rhizoma promote osteoblast differentiation and mineralization in preosteoblastic cells [20] and inhibit bone resorption in mouse osteoclasts in vitro [21]. An in vivo study indicated that the administration of Drynariae Rhizoma crude extract to 8-week-old BALB/c male mice for 5 weeks increased bone density by 6.45% and the trabecular number by 10%. Rhizoma Drynariae extracts can also induce bone formation on the margins of defects created in New Zealand white rabbit parietal bone [22]. Additionally, modern pharmacological research demonstrates that Drynariae Rhizoma has many other biological activities, including immune promotion [23], anti-inflammatory effects [24], neuroprotection [25] and treatment of hyperlipidemia, arteriosclerosis and rheumatism [26]. However, the effect of Drynariae Rhizoma on prolonged disuse or non-weight-bearing conditions on bone has not yet been investigated.

Our previous study demonstrated that total flavonoids of Drynariae Rhizoma effectively improve bone properties in ovariectomized rats and exert estrogen-like activities in osteoblast-like (MC3T3-E1) cells (data not shown). Although evidence supports the osteoprotective effect of Drynariae Rhizoma in ovariectomized rats [27], there is no direct evidence confirming its effect on disuse-induced osteoporosis. The hindlimb unloading tail-suspension (HLU) model is extensively utilized as a simulated mechanical inactivity model under reduced weight bearing or disuse [28]. In rats, hindlimb unloading results in a significant bone response, including the loss of bone mass and alterations in bone architecture and biochemical markers of bone turnover, which are similar to the response in humans [29]. Herbal extracts instead of a single component agent are frequently used in proprietary traditional Chinese medicine products; therefore, our study investigated the osteo-protective effect of total flavonoids, the main active ingredients in Drynariae Rhizoma, on bone mineral density and characterized the microarchitecture of trabecular bone using HLU rats.

## 2. Results

### 2.1. Body Weights

**Figure 1:**
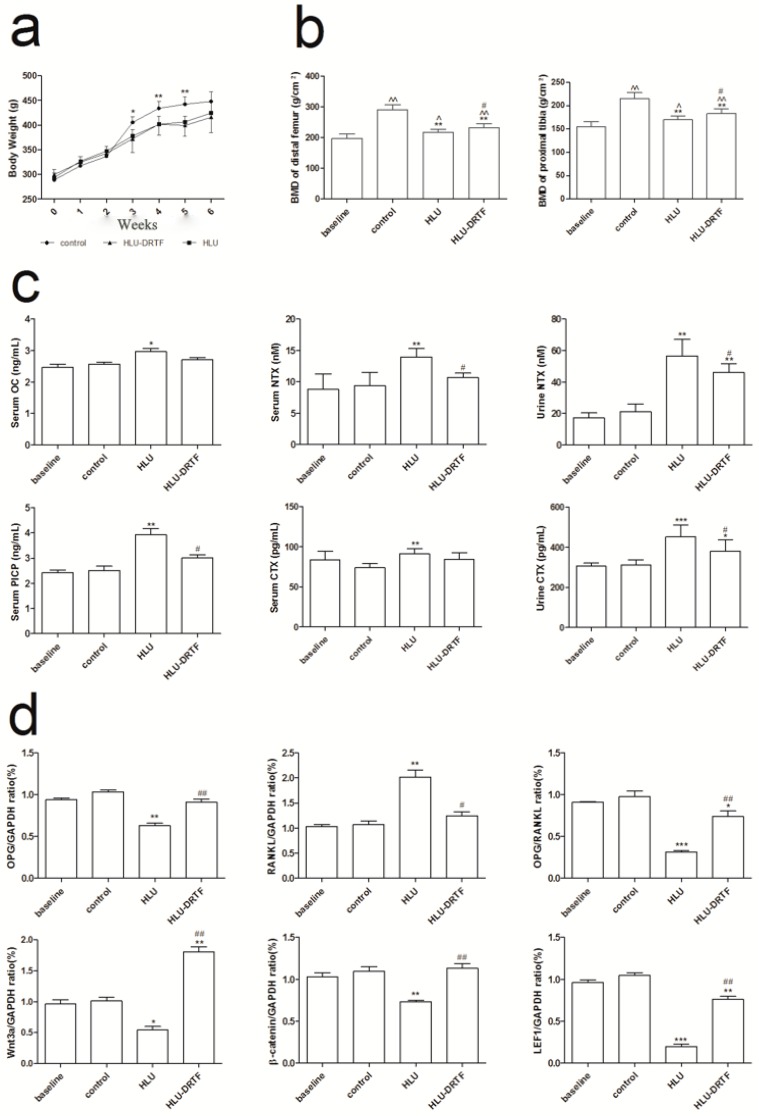
Effects of 4 weeks of treatment with total Drynariae Rhizoma flavonoids (DRTF). (**a**) The body weights within various treatment groups. Group size is *n* = 7; (**b**) Bone mineral density (BMD) of distal femur and proximal tibia within various treatment groups, *n* = 7; (**c**) Biochemical parameters in rat serum and urine within various treatment groups, *n* = 7; (**d**) The mRNA levels of *OPG*, *RANKL*, ratio of *OPG/RANKL, Wnt3a*, *β-catenin* and *LEF1* in tibiae, *n* = 3. Values are presented by means ± standard deviation (SD), ^^ *p* < 0.01 vs. baseline, ^ *p* < 0.05 vs. baseline, *** *p* < 0.001 vs. control, ** *p* < 0.01 vs. control, * *p* < 0.05 vs. control, ^##^ *p* < 0.01 vs. HLU, ^#^ *p* < 0.05 vs. HLU.

After 6-week administration, no significant differences were observed in the initial and final body weights among all treatment groups. The trends of body weights changes in all groups were plotted versus time (Figure 1a). The body weights of rats in all groups showed similar increasing trends during the first two weeks. After the tail-suspension started (second week), the body weights in the HLU and hindlimb unloading treated with total Drynariae Rhizoma flavonoids (HLU–DRTF) groups displayed no significant differences, but these two groups showed significant decreases in weight compared with the control group in the following three weeks, and they recovered with time in the sixth week.

### 2.2. BMD Evaluation

BMD changes after various treatments are shown in Figure 1b. Compared with baseline rats, the BMD of control, HLU and HLU–DRTF groups were increased (*p* < 0.05 or *p* < 0.01), whereas tail-suspension induced significant loss of BMD in both the distal femur (*p* < 0.01) and proximal tibia (*p* < 0.01) compared to controls. Rats treated with DRTF had increased BMD values compared with HLU rats (*p* < 0.05).

### 2.3. Biochemistry Assay

Results of serum and urinary biochemistry measurements are shown in Figure 1c. After HLU treatment, serum levels of C-terminal cross-linked telopeptides of type I collagen (CTX), N-terminal cross-linked telopeptides of type I collagen (NTX), C-terminal propeptide of type I procollagen (PICP) and osteocalcin (OC) and urinary levels of NTX and CTX were significantly enhanced (*p* < 0.05, *p* < 0.01 or *p* < 0.001). Compared with HLU rats, treatment with DRTF significantly suppressed the HLU-induced elevation in the serum concentration of NTX (*p* < 0.05) and PICP (*p* < 0.05). In addition, the HLU-induced increase in the urinary levels of CTX (*p* < 0.05) and NTX (*p* < 0.05) were clearly reduced by DRTF administration.

### 2.4. Bone Microarchitecture

**Figure 2:**
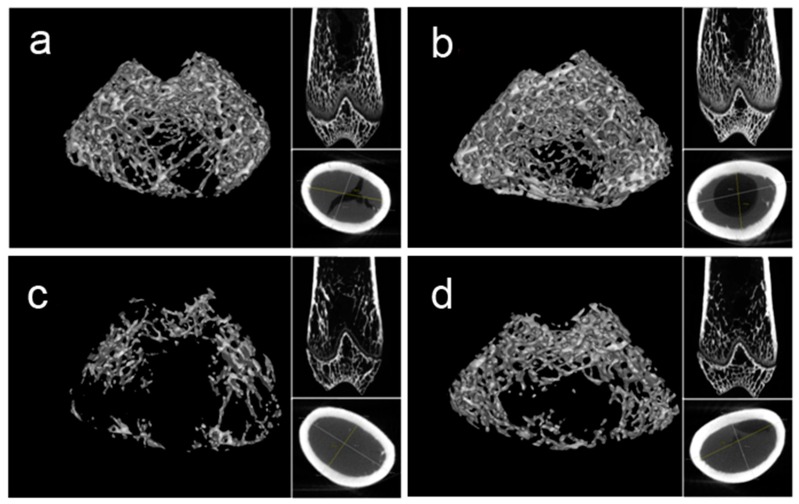
Representative three-dimensional images of trabecular bone within the distal femoral metaphyseal region in various treatment groups: (**a**) baseline; (**b**) control; (**c**) HLU; and (**d**) HLU–DRTF.

Representative three-dimensional (3D) images of trabecular bone are shown in Figure 2. The trabecular microarchitecture of the rat femur was analyzed by microCT and the results are presented in Table 1. The trabecular thickness and cortical thickness of the control group was increased compared to baseline, indicating that the skeleton grows in the normal environment over 4 weeks. Compared with the control group, tail-suspension significantly decreased bone volume/tissue volume (BV/TV, *p* < 0.01), trabecular thickness (Tb. Th, *p* < 0.01) and trabecular number (Tb. N, *p* < 0.01), whereas a significantly higher trabecular separation (Tb. Sp, *p* < 0.01) was observed in HLU rats. The microarchitecture parameters of BV/TV, Tb. Th and Tb. N were significantly improved by DRTF treatment (*p* < 0.05) and the Tb. Sp was significantly reduced compared with HLU rats (*p* < 0.05).

### 2.5. Biomechanical Test

The biomechanical parameters of the three-point bending test on the femur and tibia are summarized in Table 2. In rat femurs, the biomechanical parameters of maximum stress, maximum load, stiffness and energy were increased in the control compared with the baseline (*p* < 0.05 or *p* < 0.01). HLU treatment significantly decreased the maximum stress (*p* < 0.01), Young’s modulus (*p* < 0.05), and maximum load, stiffness (*p* < 0.01) and energy (*p* < 0.001) compared with the control group. Furthermore, DRTF administration significantly prevented the HLU-induced decrease in maximum stress, Young’s modulus, maximum load and stiffness (*p* < 0.05).

In rat tibiae, all biomechanical parameters we tested were increased in the control compared to baseline (*p* < 0.05 or *p* < 0.01). HLU treatment significantly decreased the maximum stress, Young’s modulus, maximum load, and energy (*p* < 0.01) as well as stiffness (*p* < 0.05) compared with the control group. However, maximum stress, Young’s modulus, maximum load and stiffness were found to be significantly higher in DRTF group than those in HLU group.

### 2.6. Bone Histomorphometry Analysis

**Figure 3:**
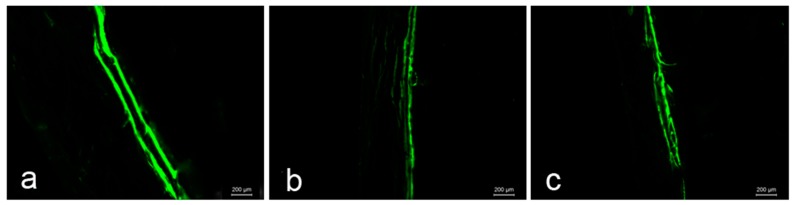
Representative fluorescence micrographs of trabecular bone sections showing green calcein labels within various treatment groups: (**a**) control; (**b**) HLU and (**c**) HLU–DRTF.

Representative fluorescent micrographs of undecalcified trabecular bone sections within various treatment groups are shown in Figure 3. New bone formation was assessed by sequential labels with fluorescent dye in undecalcified bone sections. The HLU group attained a significantly lower rate of calcification (3.19 ± 0.62 µm/d) than the control group (5.75 ± 0.43 µm/d) (*p* < 0.01). In addition, DRTF treatment increased the mineral apposition rate (MAR) in the HLU–DRTF group (4.56 ± 0.41 µm/d) compared with the HLU group (*p* < 0.05).

### 2.7. Results of OPG, RANKL Wnt3a, β-Catenin and LEF1 Gene Expression

As shown in Figure 1d, DRTF significantly increased *OPG* expression compared with the HLU group (*p* < 0.01). By contrast, *RANKL* expression was down-regulated following DRTF treatment (*p* < 0.05). The *OPG* to *RANKL* ratio was then calculated to demonstrate its effects on osteoclastogenesis and the ratio was significantly increased in the DRTF group compared with the HLU group (*p* < 0.01).

The expression of *Wnt3a*, *β-catenin* and *LEF1*, which belong to the wnt/β-catenin pathway, were detected. Our results indicate that *Wnt3a*, *β-catenin* and *LEF1* expression is down-regulated following HLU treatment (*p* < 0.05, *p* < 0.01 or *p* < 0.001), whereas DRTF treatment significantly enhanced the mRNA level of these three genes in HLU rats (*p* < 0.01).

### 2.8. Results of β-Catenin Protein Expression

**Figure 4:**
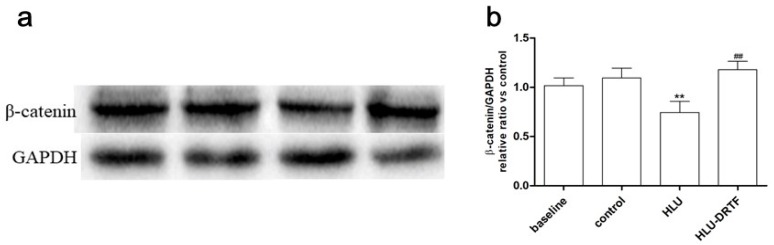
The protein expression of β-catenin in baseline, control, HLU and HLU–DRTF groups. (**a**) western blot analysis of β-catenin expressions in different groups; (**b**) “Quantity one” from Bio-Rad was used to analyze the western blotting results. The values were expressed as mean ± SD (*n* = 3), ** *p* < 0.01 vs. control, ^##^ *p* < 0.01 vs. HLU.

The protein production of β-catenin was detected using western blotting, As shown in Figure 4. The β-catenin expression is down-regulated following HLU treatment (*p* < 0.01), whereas DRTF administration significantly enhanced the protein level of β-catenin in the HLU–DRTF group (*p* < 0.01).

### 2.9. Identification of Active Compounds in DRTF

**Figure 5:**
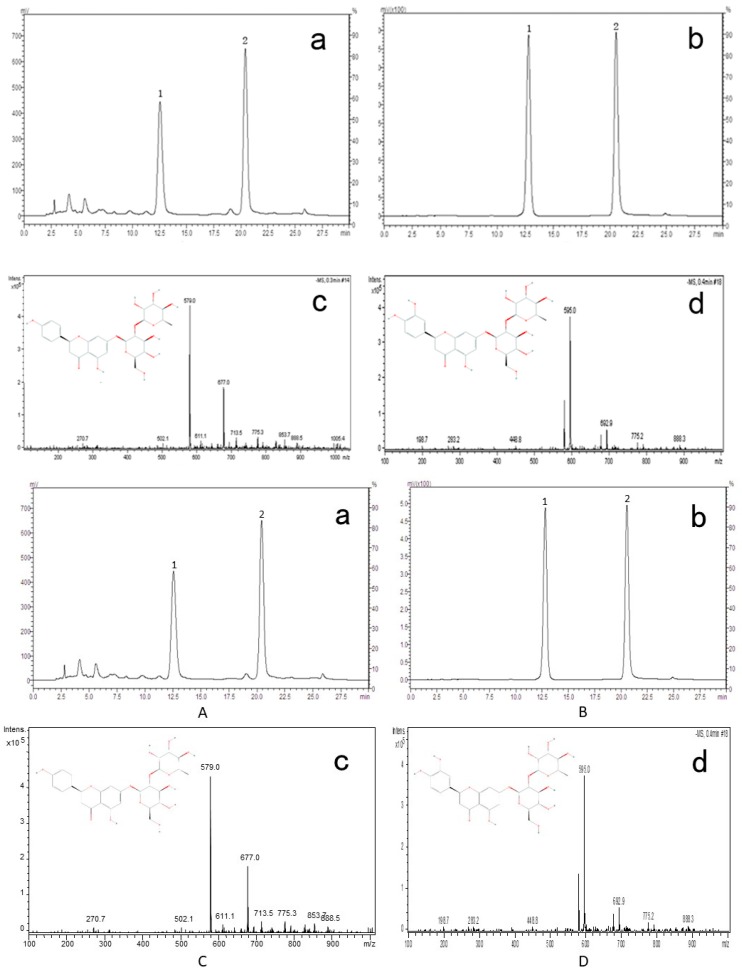
The HPLC chromatograms of DRTF (**a**) and standards (**b**); Peak 1 is neoeriocitrion, peak 2 is naringin. The primary mass spectrograms of naringin (**c**) and neoeriocitrion (**d**).

Chromatographic analysis of DRTF revealed two typical peaks numbered 1 and 2 (Figure 5a). Their retention times were 12.53 and 20.41 min, respectively. To further identify the active compound found in DRTF, we selected two standards based on the literature [30] and mass spectrometry analyses. Figure 5b is a typical chromatography profile of the two standards in which neoeriocitrion showed a high peak at retention time 12.54 min and naringin showed a high peak at retention time 20.41 min. The chemical formula of naringin is C_27_H_32_O_14_ and its molecular weight is 580.54. The chemical formula of neoeriocitrion is C_27_H_32_O_15_ and its molecular weight is 596.174. Therefore, the ESI-MS *m*/*z* for naringin is 579 [M − H]^−^ and the ESI-MS *m*/*z* for neoeriocitrion is 595 [M − H]^−^. The primary mass spectrograms of naringin and neoeriocitrion are shown in Figure 5c,d. Based on the normalization areas of the total chromatograms, the relative percentages of naringin and neoeriocitrin in DRTF were 32.92% and 43.43%, respectively.

## 3. Discussion

Due to the difficulty and high cost of conducting experiments during a space flight, a number of in vitro and in vivo ground-based models have been established to simulate aerospace environments and conditions during a space mission [29]. The HLU rat/mice model is a frequently used animal model that simulates weightless conditions [31]. Several researchers suggested that HLU may reproduce the effect of real microgravity on the musculoskeletal, cardiovascular, and immune systems of a living organism [32,33,34]. In our study, HLU treatment generated a significant decrease of BMD in the femur and tibiae. Micro CT assay also demonstrated the deterioration of trabecular bone in the femur after HLU treatment, which may be directly reflected from the 3D images or corresponding architectural parameters. The trend of changes in BMD and femur trabecular bone properties was comparable with previous studies, which demonstrates that the established HLU rat model can be used for further study [35,36]. Decreased values of biomechanical parameters also indicated the reduced strength and stiffness of the femur and tibia due to HLU treatment. HLU-induced bone loss was coupled with accelerated bone remodeling, which was evidenced by enhanced bone turnover marker levels, such as serum levels of NTX, CTX, OC, and PICP and urinary levels of NTX and CTX. These results suggest that HLU alters bone homeostasis and causes a significant increase in bone turnover rate. 

Bone adapts its mass and architecture in response to mechanical loading, and physical inactivity (i.e., spaceflight, hypokinesia) would accelerate bone microarchitecture deterioration and demineralization [37]. Although the triggers and molecular mechanisms responsible for disuse-induced deleterious effects on bone mass and strength are not fully understood, it is hypothesized that it results, in part, from the increased production of oxygen-derived free radicals and proinflammatory cytokines [38]. Drynariae Rhizoma is a well-known kidney-tonifying traditional Chinese medicine with pharmacological activities in animal experiments against osteoporosis, bone fractures, oxidative damage, and inflammation [26]. Therefore, it is highly possible that DRTF is capable of preventing weightlessness-induced bone loss.

In the current study, oral DRTF treatment for 28 continuous days during tail-suspension enhanced the femur and tibia BMD compared with HLU rats. Moreover, DRTF treatment effectively attenuated the deterioration of femoral trabecular bones. The microarchitectural properties were improved after DRTF administration, as shown by increased BV/TV, Tb. Th and Tb. N and decreased Tb. Sp. We also tested the changes in cortical bone. Compared with the baseline group, the Tb. Th and cortical thickness (Cr. Th) in distal femoral metaphysis of control group increased significantly, indicating that under normal circumstances, the bones have a certain growth after 2 weeks. However, there was no significant difference in Cr. Th between control, HLU and HLU–DRTF groups. The main reason may be that bone remodeling occurs only on the bone surface that is associated with the bone marrow and the surface area of cancellous bone is larger, accordingly, the bone remodeling in cancellous bone is more active. However, the surface area of cortical bone is smaller, which is why DRTF has a greater effect on cancellous bone and does not have much effect on cortical bone. In addition, trabecular disconnection is an independent factor in age-related skeletal failure where real termini (ReTm) may cause weakness disproportionate to tissue loss. This parameter should be pay more attention to in further research [39]. Although we observed some positive effects from DRTF treatments on trabecular bone micro-architectural properties, it is difficult to restore the deteriorated trabecular bone [40]. Sibonga et al. estimated that recovery of approximately 94% of the bone lost in career astronauts over 4–6 months of spaceflight requires 3 years of resuming normal weight-bearing activity in 1 g. Clearly, the rate of recovery upon resuming ambulatory activity is much slower than the original rate of bone loss, emphasizing the need for trabecular bone loss prevention [7,41].

Micro CT can reflect the bone microarchitecture during scan time, but the growth of bone is dynamic. The distance of two fluorescence labeling can be marked in bone tissue by two injections of calcein, thus reflecting the MAR of bone at the interval. Our results showed that the MAR in HLU group decreased significantly compared to the control group, nevertheless, MAR increased significantly after DRTF treatment compared with the HLU group, indicating that the DRTF administration promoted the bone formation.

Similar results were generated according to a biomechanical assay via a three-point bending test. HLU treatment significantly decreased bone biomechanical parameters in the rat femur and tibia and made the long bone more fragile. DRTF administration showed a comparable effect on bone strength. Maximum load, stiffness and energy were influenced by bone material properties and by bone architecture or geometric properties, which are called structural biomechanical properties or extrinsic biomechanical properties. The actual effects of the treatment with regard to biomechanical competence can only be fully evaluated by the material biomechanical parameters. Therefore, maximum stress and Young’s modulus, which are intrinsic biomechanical properties, are corrected and generated [42]. After 4 weeks of DRTF treatment, the maximum stress, Young’s modulus, maximum load and stiffness of the femur and tibia were promoted to a certain degree, and these improvements may due to trabecular bone structure optimization.

Osteoprotegrin (OPG) and receptor activator of NF-κB ligand (RANKL) were identified as the dominant and final mediators of osteoclastogenesis [43,44]. Osteoclast differentiation from osteoclast progenitors is controlled by the action of receptor activator of NF-κB ligand, a major cytokine that is expressed by osteoblast and stromal cells [45]. Moreover, osteoblasts also secrete osteoprotegerin, a soluble RANKL decoy receptor that antagonizes the proosteoclastogenic activity of RANKL by preventing it from binding to its cognate RANK receptor present on osteoclasts and their precursor cells [46]. Thus, the relative OPG/RANKL ratio determines bone mass and strength, and high OPG levels are protective against excessive osteoclastic bone resorption. Therefore, we next characterized the relative levels of expression of *OPG* and *RANKL* in whole tibia samples of the four groups of rats. Our results verified that DRTF stimulated *OPG* and suppressed *RANKL* expression, leading to an increased expression ratio of *OPG/RANKL* in rats, which may inhibit osteoclastogenesis via modulation of the OPG/RANKL system in osteoblastic cells.

The canonical wnt/β-catenin pathway functions as a modulator of the loading response [32] and plays a critical role in normal bone and cartilage formation and bone homeostasis [47,48]. β-catenin is the central mediator of canonical wnt signaling [49]. Previous studies demonstrated the important role of Wnt/β-catenin signaling in osteocyte mechanosensation [50]. It is generally assumed to be a major signaling pathway required for mechanotransduction in bone in vivo [51]. Our data indicate that DRTF significantly increased the expression of the Wnt-responsive genes, *Wnt3a*, *β-catenin* and *LEF1*, which were down-regulated by HLU treatment. The protein production of β-catenin was also detected; the β-catenin expression is down-regulated following HLU treatment, whereas DRTF administration significantly enhanced the protein level of β-catenin in the HLU–DRTF group. The results are indicative of inhibition of wnt/β-catenin signaling upon mechanical unloading and activation by DRTF treatment. Wnt/β-catenin signaling also negatively regulates osteoclast formation by inducing the secretion of OPG by osteoblasts and leading to sequestration of RANKL by OPG and the subsequent neutralization of RANKL [52]. Taken together, these findings suggest that DRTF activates Wnt signaling in vivo from unloaded bone, in part, through the increased expression ratio of *OPG/RANKL*. If so, these changes would favor increased BMD by stimulating bone formation and reducing bone resorption.

With regard to bone turnover markers, DRTF significantly suppress the HLU-induced elevation in the serum concentration of NTX and PICP. The urinary levels of CTX and NTX were also obviously reduced by the DRTF administration. Based on the results, it can be seen that DRTF administration reduces enhanced bone remodeling by HLU. However, so far it is hard to determine which dominated between the effect of DRTF on bone resorption and bone formation. Approximately 10 components have been separated in Drynariae Rhizoma [53,54], in which naringin and neoeriocitrin are two major flavonoids with the highest concentrations in total Drynariae Rhizoma flavonoids as reported previously [30]. Naringin is considered to be a major compound of DRTF and a commonly used marker for the authentication of Drynariae Rhizoma extract, according to the Chinese Pharmacopoeia [55]. It is reported that naringin was effective in protecting against ovariectomy (OVX)-induced bone loss in mice and reducing bone resorption in rat model of alveolar bone resorption [20]. Its actions might be mediated through oestrogen-like protective effects in osteoblastic cells and bone [56,57]. In addition, naringin was found to be able to synergistically enhance the secretion of OPG by osteoblasts in vitro and inhibit the formation of osteoclasts [58]. However, neoeriocitrin, a new compound isolated from Drynaria Rhizome, appears to have greater activity than naringin on proliferation and osteogenic differentiation in MC3T3-E1 cells [59]. These results confirm that the preventive effect of DRTF on bone loss might be related to the effects of two major flavonoids, naringin and neoeriocitrin, on the osteoblasts. Additional studies are ongoing, examining the osteo-protective effects of naringin and neoeriocitrin in the HLU rat model and assessing whether they produce anti-osteoporotic effects similar to those of Drynariae Rhizoma. Further experiments are warranted to reveal the potential mechanisms behind the osteogenetic effect of DRTF and other constituents in DRTF.

## 4. Materials and Methods 

### 4.1. Animals and Treatments 

Male Sprague-Dawley rats (SIPPR-BK Experimental Animal Ltd., Xi’an, China) aged 12 weeks were supplied by the Animal Center of the Fourth Military Medical University (Xi’an, China). The rats were housed in a facility maintained at 24 °C and with a 12/12 h light/dark cycle. During the experimental period, the rats were fed standard rodent chow (Animal Center of the Key Laboratory for Space Bioscience and Biotechnology, Xi’an, China) that contained 0.9% calcium and 0.7% phosphate, and filtered water was available ad libitum. After 2 weeks of acclimatization to the housing environment, twenty-eight rats were randomized into the following four groups (*n* = 7 per group): baseline group, sacrificed before HLU (baseline); control group maintained on drinking water only (control); hindlimb unloading group maintained on drinking water (HLU); and DRTF in the drinking water at a dose of 75 mg/kg body weights/day approximately (HLU–DRTF). Animals were assigned to groups by total body BMD and body mass in a manner to minimize differences between groups at baseline. For the control group, rats were allowed to move freely with their four limbs, without hindlimb unloading. In the HLU and HLU–DRTF groups, the tails of the rats were suspended such that their hindlimbs were unloaded according to the methods of Morey–Holton with modification [28]. Dosing occurred for 4 weeks and rats underwent tail-suspension from the second week. Rat body weight was recorded every week to assess changes. All tail-suspended rats were adjusted weekly to ensure that the hindlimb paws could not touch the ground. All animal handling procedures were performed in compliance with the ‘Principles of Laboratory Animal Care’ (National Institutes of Health (NIH) publication No. 85–23, revised in 1985) and the Guidance for Care and Use of Laboratory Animals with the approval of the Experimental Biology and Medicine Institutional Ethics Committee of the Fourth Military Medical University.

### 4.2. Bone Mineral Density (BMD) Analysis

BMDs at distal femoral metaphysis and proximal tibial metaphysis were measured by dual-energy X-ray absorptiometry (DEXA) assay (Lunar Prodigy Advance DXA, GE healthcare, Madison, WI, USA) using the small laboratory animal scan mode [60]. Data were calculated automatically by purpose-designed software (enCORE 2006, GE Healthcare, Madison, WI, USA). Before measurement, rats were anesthetized by pentobarbital sodium and fixed at repeatable positioning. Total BMD was measured including the cross-sectional areas of both cortical and trabecular bones.

### 4.3. Assay for Serum and Urine Chemistry

At the end of the tail-suspension, each anesthetized rat was sacrificed by exsanguination and blood was collected by cardiac puncture. Then, the blood sample was separated by centrifugation (3000× *g* for 10 min at 4 °C) to obtain serum. Urine was collected over 24 h from fasted, individually housed rats in metabolic cages. Urine and serum samples were stored at −80 °C for biochemical determinations. Serum and urine CTX and NTX were assessed using rat ELISA kits (Sinoukbio, Beijing, China). Serum PICP and osteocalcin OC concentrations were assessed using rat ELISA kits (Biocalvin, Suzhou, China) [61].

### 4.4. Microcomputed Tomography (MicroCT) Analysis

The distal metaphysis in the right femur of each rat was scanned with an Inveon microCT (Siemens, Munich, Germany). The graphic image resolution was 1024 × 1024. Three-dimensional (3D) image data were acquired with a voxel size of 20 × 20 μm in all spatial directions with a microCT evaluation program (V5.0A) [62]. The volumes of interest (VOI), which were located 1 mm from the metaphyseal line to 2 mm (100 continuous slices) below it, were selected for data analysis. The 3D segmented images were directly used to quantify the microarchitecture with the Micro-View program (Siemens) and a multiple Intel^®^ processor-based microCT workstation. Morphological measurements were performed and the following 3D parameters were obtained by analyzing the VOIs for the trabecular bone: (1) bone volume/tissue volume (BV/TV, %); (2) trabecular number (Tb. N, 1/mm); (3) trabecular thickness (Tb. Th, mm); (4) trabecular separation (Tb. Sp, mm), and (5) cortical thickness (Cr. Th, mm) [63].

### 4.5. Biomechanical Test

Prior to mechanical testing, the left femurs and tibiae were removed from the freezer, submerged in physiological saline solution to thaw slowly, and held at room temperature on the day of testing. Then, each specimen was placed in a material testing machine on two supports separated by a distance of 20 mm and load was applied to the middle of the diaphysis, thus creating a three-point bending test. The biomechanical quality of the left femoral diaphysis was determined using an AGS-10KNG material testing machine (SHIMADU, Tokyo, Japan) at a speed of 2 mm/min until fracture occurred. The central loading point was displaced, and the load and displacement values were recorded until the specimen was broken. From the load-deformation curve, the maximum load (ultimate strength, *F*_max_), energy absorption (area under the curve, *W*_abs_) and stiffness (slope of the linear region of the curve representing elastic deformation), maximum stress (*F*_max_/cross-sectional area, *σ*_ma_*x*) and Young's modulus values (maximum slope of the stress-strain curve, *E*) were obtained [64].

### 4.6. Bone Histomorphometry

Bone histomorphometry analysis was applied to evaluate the mineral apposition rate (MAR). Briefly, rats received an intramuscular injection of calcein saline solution (10 mg/kg of body weight, Sigma-Aldrich, Saint Louis, MO, USA) at 13 and 3 days prior to necropsy to evaluate bone formation dynamics. After the animals were euthanized, the specimens were fixed in 10% formalin solution and embedded in methylmethacrylate. For each animal, a set of 5-μm nonconsecutive longitudinal sections was cut in the right femur using a circular water-cooled diamond saw (Leica microtome SP1600, Wetzlar, Germany). The sections were visualized and photographed under a fluorescence microscope (Leica M205, Wetzlar, Germany) with the excitation wavelength and emission wavelengths at 490 and 515 nm, respectively. The mean calcein double-labeling interval was determined using the Zeiss Axioplan Imaging System. The interval was divided by the administration time interval (10 days), and the mean value is the MAR (µm/d) [65].

### 4.7. Real Time RT-PCR Analysis

The whole left tibia was excised and immediately frozen in liquid nitrogen and stored at −80 °C until required. The frozen femur was put in a RNase-free mortar and pestle which contained liquid nitrogen and ground to fine powder immersed in liquid nitrogen. The frozen powder was transferred into a tube containing Trizol and total RNA was isolated according to the manufacturer’s protocols (Invitrogen, Life Technologies, Carlsbad, CA, USA). The quality and quantity of total RNA were assayed using agarose gel electrophoresis and Nanodrop ND-2000 (Thermo Fisher Scientific, Waltham, MA, USA), respectively. Complementary DNA (cDNA) were synthesized using the the PrimeScript^TM^ RT reagent kit (TaKaRa, Dalian, China). The PCR amplification was performed on a LightCycler96 Thermal Cycler (Roche, Indianapolis, IN, USA) with specific primers and SYBR Premix Ex Taq^TM^ (TaKaRa). The cycling parameters were as follows: pre-denaturation at 95 °C for 30 s, followed by 40 cycles of 95 °C for 10 s, 57 °C, 58 °C or 60 °C for 15 s, and 72 °C for 10 s. Following the PCR reaction, melting curve analysis was conducted to verify the specificity and identity of the PCR product. Ct values for the samples were normalized to those of GAPDH and the relative expression was calculated via the 2-ΔΔCt method [66,67]. All reactions were performed in triplicate. The primer sequences for the real time RT-PCR were as follows: *OPG*, sense (5′-GCTCCTGGCACCTACCTA-3′) and antisense (5′-GCACTCCTGTTTCACGGT-3′); *RANKL*, sense (5′-CATCGGGTTCCCATAAAG-3′) and antisense (5′-GAAGCAAATGTTGGCGTA-3′); *Wnt3a*, sense (5′-TCCGACTCTTGGCAGAACTT-3′) and antisense (5′-AATGGAATAGGTCCCGAACA-3′); *β-catenin*, sense: (5′-GCAGCAGCAATCTTACCT-3′) and antisense (5′-GTGAAGGACTGGGAAAAG-3′); *LEF1*, sense (5′-AGCCTGTTTATCCCATCACG-3′) and antisense (5′-TGAGGCTTCACGTGCATTAG-3′); and *GAPDH*, sense (5′-TATCGGACGCCTGGTTAC-3′) and antisense (5′-CTGTGCCGTTGAACTTGC-3′).

### 4.8. Western Blotting

The whole right tibia was immediately frozen in liquid nitrogen and stored at −80 °C until required. The frozen femur was put in a RNase-free mortar and pestle which contained liquid nitrogen and ground to fine powder immersed in liquid nitrogen. The frozen powder was transferred into a tube containing RIPA lysis solution (HEART Biological Technology Co., Ltd., Shanghai, China) and total powder lysates were centrifuged at 10,000 RPM for 10 min at 4 °C. The supernatants were collected and the total protein content was quantified with a BCA assay kit (HEART Biological Technology Co., Ltd., Shanghai, China). Then, 15 μg of total protein from each sample was separated by SDS-PAGE (10% gel) and transferred to PVDF membranes. After incubation in blocking solution (5% non-fat milk, 0.05% Tween-20 in TBS) for 2 h at room temperature, the membrane was incubated with an appropriate primary antibody against β-catenin (R&D Systems, Minneapolis, MN, USA) and GAPDH (Proteintech, Wuhan, China) at 4 °C overnight. After three washes with TBS containing 0.05% Tween-20 (TBST), the membranes were incubated with horseradish peroxidase (HRP) conjugated secondary antibody for 2 h at room temperature. Next, the immunoreaction signals were detected with the enhanced ECL chemiluminescence reagent kit (Biodragon-immunotech, Beijing, China) and exposed on the Gel Doc™ XR + System (Bio-Rad, Hercules, CA, USA). The relative protein intensities were quantified using Quantity One 1-D analysis software (Bio-Rad) and GAPDH was used as internal control.

### 4.9. Identification of Active Phytochemicals in DRTF

Phytochemicals of DRTF were identified by high performance liquid chromatography (HPLC), (LC-2010C, Shimadzu, Japan) and mass spectrometry (MS), (Esquire 6000, Bruker, Germany). A 10-μL solution was subjected to HPLC and analyzed with a UV detector (SPD-M20A, Shimadzu, Japan) at 283 nm. The mobile phase was composed of (a) methanol and (b) 0.1% acetic acid water, with a gradient elution as follows: 0~14 min, 30–35% A; 14~22 min, 35–50% A; 22~26 min, 50–35% A; 26~35 min, and 35–35% A. The flow rate of the mobile phase was 1.0 mL·min^−1^, and the temperature was maintained at 30 °C. The chromatographic column was a C18 (4.6 × 250 mm, ID, 5 μm, MERCK). The MS identification conditions were as follows: the ion source was ESI, the atomization pressure was 35 psi, the dry gas flow rate was 11 mL/min, the drying temperature was 350 °C, the mass range of mass spectrometry was 100~1000 amu, and the ion mode was negative.

A standard stock solution of each of the following components was prepared directly in methanol: naringin (Aladdin, CAS#: 10236-47-2, Batch: 23,616) andneoeriocitrin (CHROMADEX, CAS#: 13241-32-2, Batch: ASB-00005190-010). Working standard solutions containing the two compounds were prepared and diluted with methanol to the appropriate concentrations for the establishment of calibration curves. The DRTF samples were also dissolved in methanol and prepared as working solutions. The standard stock solutions and working solutions were all prepared in dark brown calibrated flasks and stored at 4 °C. The relative percentages of phytochemicals in the DRTF were calculated from the area normalization of total chromatograms using a computerized integrator.

### 4.10. Statistical Analysis

Data are presented as the mean ± standard deviation (SD). One-way ANOVAs were used to compare data from all groups and Turkey’s tests were used as a post-test to compare pairs of groups using the SPSS 16.0 statistical software (SPSS Inc., Chicago, IL, USA). Differences between means were considered statistically significant when *p*-values were <0.05.

## 5. Conclusions

This study systematically evaluated the protective effects of DRTF on HLU-induced bone loss in rats. DRTF administration prevented the HLU induced bone mass decrease and deterioration of trabecular microarchitecture, thus maintaining the structural integrity and biomechanical qualities of the bone. Further study is required to explore the underlying mechanisms. However, these data indicate DRTF may be a reasonable natural alternative for the prevention of disuse osteoporosis induced by mechanical inactivity.

## Figures and Tables

**Table 1:** The trabecular microarchitecture of rat femur within various treatment groups. BV/TV: bone volume/tissue volume; Tb. Th: trabecular thickness; Tb. N: trabecular number; Tb. Sp: trabecular separation; Cr. Th: cortical thickness.

Parameters	Baseline	Control	HLU	HLU–DRTF
BV/TV (%)	0.288 ± 0.025	0.316 ± 0.010	0.183 ± 0.016 **	0.252 ± 0.007 * ^#^
Tb. Th (mm)	0.060 ± 0.004	0.072 ± 0.002 ^	0.056 ± 0.006 **	0.068 ± 0.003 ^#^
Tb. N (1/mm)	4.243 ± 0.579	4.950 ± 0.250	1.719 ± 0.439 **	2.817 ± 0.334 ** ^#^
Tb. Sp (mm)	0.146 ± 0.024	0.128 ± 0.011	0.373 ± 0.084 **	0.252 ± 0.023 * ^#^
Cr. Th (mm)	0.447 ± 0.021	0.569 ± 0.028 ^	0.496 ± 0.054	0.508 ± 0.032

Values are mean ± SD (*n* = 3), ^ *p* < 0.05 vs. baseline, ** *p* < 0.01 vs. control, * *p* < 0.05 vs. control, ^##^ *p* < 0.01 vs. HLU, ^#^ *p* < 0.05 vs. HLU.

**Table 2:** Effects of DRTF treatment on bone biomechanical parameters in rat femoral and tibial diaphysis.

	Parameters	Baseline	Control	HLU	HLU–DRTF
**Femur**	Max-Stress (Mpa)	74.05 ± 13.25	98.86 ± 9.08 ^	70.12 ± 7.31 **	90.96 ± 9.49 ^#^
	Young’s modulus (Mpa)	1673.24 ± 120.9	1891.80 ± 179.25	1593.82 ± 103.16 *	1911.27 ± 67.80 ^#^
	Max-Load (N)	105.38 ± 10.73	145.92 ± 17.08 ^^	107.61 ± 7.05 **	128.42 ± 7.16 ^#^
	Stiffness (N/mm)	111.14 ± 15.23	168.37 ± 11.32 ^^	125.94 ± 11.12 **	159.89 ± 15.54 ^^ ^#^
	Energy (N.mm)	77.15 ± 13.15	112.42 ± 19.32 ^^	61.69 ± 3.04 ***	78.08 ± 6.85 **
**Tibia**	Max-Stress (Mpa)	98.38 ± 7.59	111.92 ± 6.67 ^	85.89 ± 4.56 **	98.95 ± 4.43 * ^#^
	Young’s modulus (Mpa)	1548.24 ± 54.61	1791.80 ± 97.91 ^	1418.82 ± 87.75 **	1736.27 ± 173.61 ^##^
	Max-Load (N)	55.03 ± 5.95	72.45 ± 8.27 ^^	54.85 ± 3.24 **	69.43 ± 2.78 ^#^
	Stiffness (N/mm)	77.50 ± 10.12	97.21 ± 14.20 ^	72.99 ± 4.44 *	88.48 ± 3.43 ^#^
	Energy (N.mm)	61.32 ± 3.24	78.55 ± 7.49 ^^	61.22 ± 3.78 **	66.82 ± 2.59 *

Values are mean ± SD (*n* = 7), ^^ *p* < 0.01 vs. baseline, ^ *p* < 0.05 vs. baseline, *** *p* < 0.001 vs. control, ** *p* < 0.01 vs. control, * *p* < 0.05 vs. control, ^##^ *p* < 0.01 vs. HLU, ^#^ *p* < 0.05 vs. HLU.

## Abbreviations

d	day
DRTF	total flavonoids of Drynariae Rhizoma
OPG	osteocalcin
OVX	ovariectomy
RANKL	receptor activator of NF-κB ligand
BMD	bone mineral density
NTX-Ι	N-terminal cross-linked telopeptides of type I collagen
CTX-Ι	C-terminal cross-linked telopeptides of type I collagen
OC	osteocalcin
PICP	C-terminal propeptide of type I procollagen
BV/TV	bone volume/tissue volume
Tb. N	trabecular number
Tb. Th	trabecular thickness
Tb. Sp	trabecular separation
Cr. Th	cortical thickness
NIH	National Institutes of Health

## References

[1] Blaber E.A., Dvorochkin N., Lee C., Alwood J.S., Yousuf R., Pianetta P., Globus R.K., Burns B.P., Almeida E.A. (2013). Microgravity induces pelvic bone loss through osteoclastic activity, osteocytic osteolysis, and osteoblastic cell cycle inhibition by cdkn1a/p21. PLoS ONE.

[2] Fitts R.H., Riley D.R., Widrick J.J. (2001). Functional and structural adaptations of skeletal muscle to microgravity. J. Exp. Biol..

[3] Chowdhury B., Seetharam A., Wang Z., Liu Y., Lossie A.C., Thimmapuram J., Irudayaraj J. (2016). A study of alterations in DNA epigenetic modifications (5mc and 5hmc) and gene expression influenced by simulated microgravity in human lymphoblastoid cells. PLoS ONE.

[4] Paulsen K., Thiel C., Timm J., Schmidt P.M., Huber K., Tauber S., Hemmersbach R., Seibt D., Kroll H., Grote K.-H. (2010). Microgravity-induced alterations in signal transduction in cells of the immune system. Acta Astronaut..

[5] Nicolas M., Sandal G.M., Weiss K., Yusupova A. (2013). Mars-105 study: Time-courses and relationships between coping, defense mechanisms, emotions and depression. J. Environ. Psychol..

[6] Shackelford L.C., LeBlanc A.D., Driscoll T.B., Evans H.J., Rianon N.J., Smith S.M., Spector E., Feeback D.L., Lai D. (2004). Resistance exercise as a countermeasure to disuse-induced bone loss. J. Appl. Physiol. (Bethesda, Md.: 1985).

[7] Sibonga J.D., Evans H.J., Sung H.G., Spector E.R., Lang T.F., Oganov V.S., Bakulin A.V., Shackelford L.C., Leblanc A.D. (2007). Recovery of spaceflight-induced bone loss: Bone mineral density after long-duration missions as fitted with an exponential function. Bone.

[8] Hargens A.R., Bhattacharya R., Schneider S.M. (2013). Space physiology vi: Exercise, artificial gravity, and countermeasure development for prolonged space flight. Eur. J. Appl. Physiol..

[9] Chowdhury P., Soulsby M. (2002). Lipid peroxidation in rat brain is increased by simulated weightlessness and decreased by a soy-protein diet. Ann. Clin. Lab. Sci..

[10] Peng S., Zhang G., Zhang B.T., Guo B., He Y., Bakker A.J., Pan X., Zhen W., Hung L., Qin L. (2013). The beneficial effect of icaritin on osteoporotic bone is dependent on the treatment initiation timing in adult ovariectomized rats. Bone.

[11] Zhang Z.K., Li J., Liu J., Guo B., Leung A., Zhang G., Zhang B.T. (2016). Icaritin requires phosphatidylinositol 3 kinase (pi3k)/akt signaling to counteract skeletal muscle atrophy following mechanical unloading. Sci. Rep..

[12] Durbin S.M., Jackson J.R., Ryan M.J., Gigliotti J.C., Alway S.E., Tou J.C. (2012). Resveratrol supplementation influences bone properties in the tibia of hindlimb-suspended mature fisher 344 x brown norway male rats. Appl. Physiol. Nutr. Metab..

[13] Habold C., Momken I., Ouadi A., Bekaert V., Brasse D. (2011). Effect of prior treatment with resveratrol on density and structure of rat long bones under tail-suspension. J. Bone Miner. Metab..

[14] Kong X.H., Niu Y.B., Song X.M., Zhao D.D., Wang J., Wu X.L., Zhang R., Mei Q.B. (2012). Astragaloside II induces osteogenic activities of osteoblasts through the bone morphogenetic protein-2/mapk and smad1/5/8 pathways. Int. J. Mol. Med..

[15] Xu Y.X., Wu C.L., Wu Y., Tong P.J., Jin H.T., Yu N.Z., Xiao L.W. (2012). Epimedium-derived flavonoids modulate the balance between osteogenic differentiation and adipogenic differentiation in bone marrow stromal cells of ovariectomized rats via wnt/beta-catenin signal pathway activation. Chin. J. Integr. Med..

[16] Niu Y.B., Li Y.H., Kong X.H., Zhang R., Sun Y., Li Q., Li C., Liu L., Wang J., Mei Q.B. (2012). The beneficial effect of radix dipsaci total saponins on bone metabolism in vitro and in vivo and the possible mechanisms of action. Osteoporos. Int..

[17] Zhang R., Liu Z.G., Li C., Hu S.J., Liu L., Wang J.P., Mei Q.B. (2009). Du-zhong (*Eucommia ulmoides* oliv.) cortex extract prevent ovx-induced osteoporosis in rats. Bone.

[18] Chen Z.Q. (2004). Ben Cao Shi Yi.

[19] Huang Y., Liu X., Zhao L., Li F., Xiong Z. (2014). Kidney tissue targeted metabolic profiling of glucocorticoid-induced osteoporosis and the proposed therapeutic effects of rhizoma drynariae studied using uhplc/ms/ms. Biomed. Chromatogr..

[20] Chen L.L., Lei L.H., Ding P.H., Tang Q., Wu Y.M. (2011). Osteogenic effect of drynariae rhizoma extracts and naringin on mc3t3-e1 cells and an induced rat alveolar bone resorption model. Arch. Oral Biol..

[21] Jeong J.C., Kang S.C., Jeong C.W., Kim H.M., Lee Y.C., Chang Y.C., Kim C.H. (2003). Inhibition of drynariae rhizoma extracts on bone resorption mediated by processing of cathepsin k in cultured mouse osteoclasts. Int. Immunopharmacol..

[22] Wong R.W., Rabie B., Bendeus M., Hagg U. (2007). The effects of rhizoma curculiginis and rhizoma drynariae extracts on bones. Chin. Med..

[23] An H.J., Lee G.G., Lee K.T. (2012). Drynariae rhizoma increases immune response in mice. Nat. Prod. Commun..

[24] Anuja G.I., Latha P.G., Suja S.R., Shyamal S., Shine V.J., Sini S., Pradeep S., Shikha P., Rajasekharan S. (2010). Anti-inflammatory and analgesic properties of *Drynaria quercifolia* (L.) j. Smith. J. Ethnopharmacol..

[25] Wang W., Li H., Yu J., Hong M., Zhou J., Zhu L., Wang Y., Luo M., Xia Z., Yang Z.J. (2016). Protective effects of chinese herbal medicine rhizoma drynariae in rats after traumatic brain injury and identification of active compound. Mol. Neurobiol..

[26] Sung Y.Y., Kim D.S., Yang W.K., Nho K.J., Seo H.S., Kim Y.S., Kim H.K. (2012). Inhibitory effects of drynaria fortunei extract on house dust mite antigen-induced atopic dermatitis in nc/nga mice. J. Ethnopharmacol..

[27] Lee Y.E., Liu H.C., Lin Y.L., Liu S.H., Yang R.S., Chen R.M. (2014). *Drynaria fortunei* j. Sm. Improves the bone mass of ovariectomized rats through osteocalcin-involved endochondral ossification. J. Ethnopharmacol..

[28] Morey-Holton E.R., Globus R.K. (1998). Hindlimb unloading of growing rats: A model for predicting skeletal changes during space flight. Bone.

[29] Vico L., Hinsenkamp M., Jones D., Marie P.J., Zallone A., Cancedda R. (2001). Osteobiology, strain, and microgravity. Part ii: Studies at the tissue level. Calcif. Tissue Int..

[30] Li Y.-B., Meng F.-H., Pan X.-F., Xiong Z.-L., Li F.-M. (2006). Hplc determination of neoeriocitrin and naringin in rhizoma drynariae. Chin. J. Pharm. Anal..

[31] Wimalawansa S.M., Wimalawansa S.J. (1999). Simulated weightlessness-induced attenuation of testosterone production may be responsible for bone loss. Endocrine.

[32] Felix K., Wise K., Manna S., Yamauchi K., Wilson B.L., Thomas R.L., Kulkarni A., Pellis N.R., Ramesh G.T. (2004). Altered cytokine expression in tissues of mice subjected to simulated microgravity. Mol. Cell. Biochem..

[33] Morey-Holton E., Globus R.K., Kaplansky A., Durnova G. (2005). The hindlimb unloading rat model: Literature overview, technique update and comparison with space flight data. Adv. Space Biol. Med..

[34] Sonnenfeld G., Morey E.R., Williams J.A., Mandel A.D. (1982). Effect of a simulated weightlessness model on the production of rat interferon. J. Interferon Res..

[35] Siu W.S., Wong H.L., Lau C.P., Shum W.T., Wong C.W., Gao S., Fung K.P., Lau C.B., Hung L.K., Ko C.H. (2013). The effects of an antiosteoporosis herbal formula containing epimedii herba, ligustri lucidi fructus and psoraleae fructus on density and structure of rat long bones under tail-suspension, and its mechanisms of action. Phytother. Res..

[36] Bloomfield S.A., Allen M.R., Hogan H.A., Delp M.D. (2002). Site- and compartment-specific changes in bone with hindlimb unloading in mature adult rats. Bone.

[37] Oganov V.S. (2004). Modern analysis of bone loss mechanisms in microgravity. J. Gravitat. Physiol..

[38] Smith B.J., Lucas E.A., Turner R.T., Evans G.L., Lerner M.R., Brackett D.J., Stoecker B.J., Arjmandi B.H. (2005). Vitamin e provides protection for bone in mature hindlimb unloaded male rats. Calcif. Tissue Int..

[39] Aaron J.E., Shore P.A., Itoda M., Morrison R.J., Hartopp A., Hensor E.M., Hordon L.D. (2015). Mapping trabecular disconnection “hotspots” in aged human spine and hip. Bone.

[40] Devareddy L., Khalil D.A., Smith B.J., Lucas E.A., Soung do Y., Marlow D.D., Arjmandi B.H. (2006). Soy moderately improves microstructural properties without affecting bone mass in an ovariectomized rat model of osteoporosis. Bone.

[41] Laib A., Kumer J.L., Majumdar S., Lane N.E. (2001). The temporal changes of trabecular architecture in ovariectomized rats assessed by microct. Osteoporos. Int..

[42] Turner C.H., Burr D.B. (1993). Basic biomechanical measurements of bone: A tutorial. Bone.

[43] Zhang Z., Song C., Fu X., Liu M., Li Y., Pan J., Liu H., Wang S., Xiang L., Xiao G.G. (2014). High-dose diosgenin reduces bone loss in ovariectomized rats via attenuation of the rankl/opg ratio. Int. J. Mol. Sci..

[44] Bord S., Ireland D.C., Beavan S.R., Compston J.E. (2003). The effects of estrogen on osteoprotegerin, rankl, and estrogen receptor expression in human osteoblasts. Bone.

[45] Vaananen H.K., Laitala-Leinonen T. (2008). Osteoclast lineage and function. Arch. Biochem. Biophys..

[46] Boyce B.F., Xing L. (2008). Functions of rankl/rank/opg in bone modeling and remodeling. Arch. Biochem. Biophys..

[47] Kramer I., Halleux C., Keller H., Pegurri M., Gooi J.H., Weber P.B., Feng J.Q., Bonewald L.F., Kneissel M. (2010). Osteocyte wnt/beta-catenin signaling is required for normal bone homeostasis. Mol. Cell. Biol..

[48] Lara-Castillo N., Kim-Weroha N.A., Kamel M.A., Javaheri B., Ellies D.L., Krumlauf R.E., Thiagarajan G., Johnson M.L. (2015). In vivo mechanical loading rapidly activates beta-catenin signaling in osteocytes through a prostaglandin mediated mechanism. Bone.

[49] Zahoor M., Cha P.H., Min do S., Choi K.Y. (2014). Indirubin-3′-oxime reverses bone loss in ovariectomized and hindlimb-unloaded mice via activation of the wnt/beta-catenin signaling. J. Bone Miner. Res..

[50] Bonewald L.F., Johnson M.L. (2008). Osteocytes, mechanosensing and wnt signaling. Bone.

[51] Robinson J.A., Chatterjee-Kishore M., Yaworsky P.J., Cullen D.M., Zhao W., Li C., Kharode Y., Sauter L., Babij P., Brown E.L. (2006). Wnt/beta-catenin signaling is a normal physiological response to mechanical loading in bone. J. Biol. Chem..

[52] Krishnan V., Bryant H.U., Macdougald O.A. (2006). Regulation of bone mass by wnt signaling. J. Clin. Investig..

[53] Shang Z.P., Zhao Q.C., Tan J.J., Yang L., Yan M., Shi G.B. (2010). Chemical constituents from rhizomes of drynaria fortunei. Pract. Pharm. Clin. Rem..

[54] Gao Y., Wang X.L., Wang N.L., Yao X.S. (2008). Chemical constituents from drynaria fortunei. Chin. J. Med. Chem..

[55] National Pharmacopoeia Committee (2015). Pharmacopoeia of the People’s Republic of China.

[56] Pang W.Y., Wang X.L., Mok S.K., Lai W.P., Chow H.K., Leung P.C., Yao X.S., Wong M.S. (2010). Naringin improves bone properties in ovariectomized mice and exerts oestrogen-like activities in rat osteoblast-like (umr-106) cells. Br. J. Pharmacol..

[57] Wong K.C., Pang W.Y., Wang X.L., Mok S.K., Lai W.P., Chow H.K., Leung P.C., Yao X.S., Wong M.S. (2013). Drynaria fortunei-derived total flavonoid fraction and isolated compounds exert oestrogen-like protective effects in bone. Br. J. Nutr..

[58] Xu T., Wang L., Tao Y., Ji Y., Deng F., Wu X.H. (2016). The function of naringin in inducing secretion of osteoprotegerin and inhibiting formation of osteoclasts. Evid. Based Complement. Altern. Med..

[59] Li L., Zeng Z., Cai G. (2011). Comparison of neoeriocitrin and naringin on proliferation and osteogenic differentiation in mc3t3-e1. Phytomedicine.

[60] Pastoureau P., Chomel A., Bonnet J. (1995). Specific evaluation of localized bone mass and bone loss in the rat using dual-energy X-ray absorptiometry subregional analysis. Osteoporos. Int..

[61] Delmas P.D., Eastell R., Garnero P., Seibel M.J., Stepan J. (2000). The use of biochemical markers of bone turnover in osteoporosis. Committee of scientific advisors of the international osteoporosis foundation. Osteoporos. Int..

[62] Laib A., Barou O., Vico L., Lafage-Proust M.H., Alexandre C., Rugsegger P. (2000). 3d micro-computed tomography of trabecular and cortical bone architecture with application to a rat model of immobilisation osteoporosis. Med. Biol. Eng. Compt..

[63] Marinozzi F., Bini F., Marinozzi A., Zuppante F., De Paolis A., Pecci R., Bedini R. (2013). Technique for bone volume measurement from human femur head samples by classification of micro-ct image histograms. Annali dell’Istituto Superiore di Sanita.

[64] Ederveen A.G., Spanjers C.P., Quaijtaal J.H., Kloosterboer H.J. (2001). Effect of 16 months of treatment with tibolone on bone mass, turnover, and biomechanical quality in mature ovariectomized rats. J. Bone Miner. Res..

[65] Dempster D.W., Compston J.E., Drezner M.K., Glorieux F.H., Kanis J.A., Malluche H., Meunier P.J., Ott S.M., Recker R.R., Parfitt A.M. (2013). Standardized nomenclature, symbols, and units for bone histomorphometry: A 2012 update of the report of the asbmr histomorphometry nomenclature committee. J. Bone Miner. Res..

[66] Lau W.S., Chan R.Y., Guo D.A., Wong M.S. (2008). Ginsenoside rg1 exerts estrogen-like activities via ligand-independent activation of eralpha pathway. J. Steroid Biochem. Mol. Biol..

[67] Chang S., Chen W., Yang J. (2009). Another formula for calculating the gene change rate in real-time rt-pcr. Mol. Biol. Rep..

